# Attention deficit hyperactivity disorder symptom self-report in adults in Kenya and its associated risk factors, an analysis from a household survey in a demographic surveillance site

**DOI:** 10.1017/gmh.2015.14

**Published:** 2015-07-29

**Authors:** R. Jenkins, C. Othieno, L. Ongeri, B. Ogutu, P. Sifuna, J. Mboroki, R. Omollo

**Affiliations:** 1 Health Services and Population Research Department, Institute of Psychiatry, Kings College London, UK; 2 Departmenet Psychiatry, University of Nairobi, Nairobi, Kenya; 3 Kenya Medical Research Institute, Centre for Clinical Research, Nairobi, Kenya; 4 Kombewa Health and Demographic Surveillance Site, Kisumu, Kenya; 5 Kenya Medical Training Centre, Mental Health, Nairobi, Kenya

**Keywords:** ADHD, other, prevalence, risk factors, surveillance site

## Abstract

**Background.:**

There have been no household surveys of adult attention deficit and hyperactivity disorder (ADHD) in Kenya, and only one in sub-Saharan Africa.

**Methods.:**

Data on ADHD was used from a household survey of mental disorders and their associated risk factors conducted in Maseno area (population 70 805), near Lake Victoria in Kenya, using a demographic surveillance site as the sample frame, as part of a wider survey of mental health, malaria and immunity A total of 1190 households were selected, and 1158 adult participants consented to the study while 32 refused to participate in the study interviews, giving a response rate of 97.3%. ADHD symptoms were assessed with the WHO Adult ADHD Self-Report Scale (ASRS) Screener.

**Results.:**

This survey found that the overall prevalence of ADHD using the ASRS was 13.1%. This suggests a high level of ADHD in the Kenyan population which needs to be further investigated for its impact on adult mental health. In the adjusted analysis, increased odds ratios (ORs) were found in those with higher assets (OR 1.7, *p* = 0.023), those with life events (OR 2.4, *p* = 0.001 for those with 2–3 life events and OR 2.6, *p* < 0.001 for those with 4 or more life events), and those with common mental disorders (OR 2.3, *p* = 0.001).

**Conclusion.:**

The study demonstrates the magnitude of ADHD symptoms as a public health issue, relevant for health worker training, and the importance of further research into its prevalence in adults and associated risk factors.

## Introduction

Attention deficit hyperactivity disorder (ADHD) is a developmental disorder characterised by attentional problems, impulsive behaviour and hyperactivity, and symptoms starting in childhood (Biederman, 1998), but which is now known can persist into adolescence and adulthood (Mannuzza *et al*. 1993; Cumyn *et al*. 2007). The previous age criterion of diagnostic and statistical manual of mental disorders (DSM)-IV that symptoms should have started before the age of 7 has now been extended in DSM-V which makes more explicit references to the progression of ADHD into adulthood. Pooled prevalence of ADHD in children and adolescents combined has been estimated to be 5%, and around 3% in adolescents (Polanczyk *et al*. 2007). The estimated prevalence of adult ADHD in the USA is 4.4%, and is more common in males, previously married, unemployed and is associated with substantial role impairment (Kessler *et al*. 2006). A recent meta-analysis of epidemiological studies of ADHD (all conducted in high income countries) estimated a prevalence of 2.5%, declining with increasing age, and more common in men (Simon *et al*. 2009). There is thought to be substantial under-diagnosis or misdiagnosis of affected people (Kooij *et al*. 2010), and there have been calls to expand the research agenda on this condition in adulthood (Ramos-Quiroga *et al*. 2014).

The only previous African epidemiological survey of ADHD in a general adult population using a screening questionnaire was conducted in Nigeria in adults aged 18–52, using the Berkely Adult ADHD rating scale and found a population prevalence of 12.2% (Okhakume & Oluwefaemi, 2014). Therefore the opportunity was taken to conduct a study of the adult prevalence of ADHD in Kenya as part of a wider project to examine the associations between mental disorder, malaria and immunity, the results of which will be reported elsewhere.

Kenya is in sub-Saharan Africa, and was a low income country until 2014 when it became a lower middle income country, because Kenya's gross domestic product (GDP) per capita rose from US$399 in 2000 to US$ 1040.55 in 2013, although poverty levels remain high at 45.9% (UNDP, 2013). Kenya's population is growing rapidly by about 1 million a year from 10 million in 1969 to an estimated 43.2.million in 2013(extrapolated from the 2009 census), (Kenya Bureau of Statistics, 2011) with nearly 50% aged under 15. Life expectancy at birth is 57 years, the adult literacy rate is 87%, the infant mortality rate is 55 per 1000 live births, the maternal mortality rate is 490 per 100 000 live births and HIV prevalence is currently estimated at 5.6% (Kimanga *et al*. 2014). There is rapid urbanisation, the agricultural sector is highly inefficient and the food supply is vulnerable to catastrophic drought and floods.

Nyanza Province, where the study was conducted, has relatively high levels of unplanned pregnancies (53%), deaths of children under 5 (14.9%), spousal abuse (60%) (United Nations, 2007) and considerably higher prevalence compared with the rest of Kenya of HIV of 17.7% in women and 14.1% in men (Oluoch *et al*. 2011), all of which socioeconomic and health challenges may impact on the mental health of the population.

The research was conducted as part of an overall collaborative programme of work between the Kenya Ministry of Health and the UK Institute of Psychiatry, Kings College London over the last 15 years, (including collaborations with the Kenya Medical Training College (KMTC), the Kenya Medical Research Institute (KEMRI),the Kenya Psychiatric Association and Great Lakes University) comprising situation appraisal (Kiima *et al*. 2004; Muga & Jenkins 2008*a*; Mugawebster & Jenkins, 2010; Jenkins *et al*. 2012*a*, *b*), studies of traditional healers (Okonji *et al*. 2008), community health workers (Kiima *et al*. 2009), district health workers (Muga & Jenkins 2008*b*), policy development (Kiima & Jenkins, 2010), primary care training (Jenkins *et al*. 2010*a*, *b*) and its evaluation (Jenkins *et al*. 2013*a*, *b*, *c*; Othieno *et al*. 2013).

## Methods

Data were used from a cross sectional community study, of adults living at home in a demographic surveillance site in Nyanza province, near Lake Victoria in Kenya, in order to examine the prevalence rate of ADHD self-reported symptoms and associated risk factors.

### Study population

The sample frame is a subdivision in an endemic area for malaria in Kenya, namely Maseno area in Kisumu county, Nyanza Province in western Kenya, Maseno has a population of 70 805 (Sifuna *et al*. 2014). Females constitute 53% of the population. The mean household size is four people per household with a population density of about 374 people/km^2^. The population is largely young with a mean age of 23 years. The population 0–14 years constitutes 46%, ages 15–64 years constitute 49% and ages 65 + years constitute 5%.

The population is primarily black African, and the languages spoken are Luo (which is the predominant ethnic group), Kiswahili and English. The area is largely rural, with most residents living in villages, which are a loose conglomeration of family compounds near a garden plot and grazing land. The majority of the houses are mud-walled with either grass thatched or corrugated iron-sheet roofs. Water is sourced mainly from community wells (40%), local streams (43%) and the lake (5%) for those mostly living on the shores of Lake Victoria (Sifuna *et al*. 2014). Most water sources are not chlorinated. Subsistence farming, animal husbandry and fishing are the main economic activities in the area. Malaria is holoendemic in this area, and transmission occurs throughout the year. The ‘long rainy season’ from late March to May produces intense transmission from April to August. The ‘short rainy season’ from October to December produces another, somewhat less intense, transmission season from November to January.

## Study site

### Study participants

**Fig. 1.**
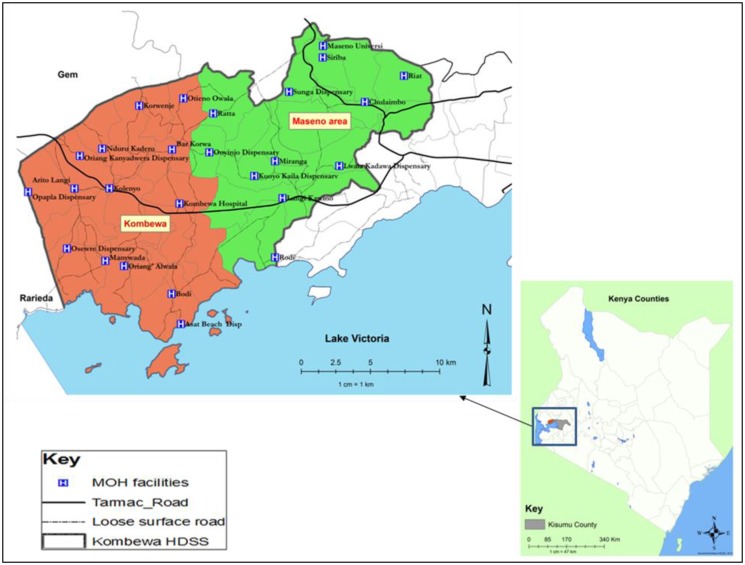
Location of the study site.

The study sample was selected from Maseno Area within Kisumu County, western Kenya (see Fig. 1). Maseno Area is sub-divided into four locations, 17 sub-locations and 184 enumeration areas (villages) based on mapping work done earlier by the Kombewa Health and Demographic Surveillance System (Kombewa HDSS) run by the KEMRI/Walter Reed Project. The Kombewa HDSS is a longitudinal population registration system set up to monitor the evolving health and demographic problems of the study population in Kombewa and Maseno areas (Sifuna *et al*. 2014). Some villages with less than 50 households were merged together to create new enumeration areas. A random sample of 7 households was drawn from each enumeration area, to give a projected sample of 1190 households, which with an estimated response rate of 85% would give a total sample size of 1010. Village maps were used to assign households and guide the research assistants during the survey. Using the Kish Grid Method, one individual was selected from each of the sampled household. The demographics and reasons for the refusal were recorded in notebooks by the Research assistants. A total of 1190 households were visited.

### Study procedures

Meetings were held with community leaders to explain the purpose of the survey, and answer questions. The heads of the sampled households, and then the identified participants in the survey were approached in their own homes for informed written and witnessed consent to the interview. The interview was administered by one of a group of 20 research assistants using a personal digital assistant (PDA), on which the interview questions were programmed and responses were recorded. The research assistants received a 5 day training course, and were supervised in the field by a field manager.

The participants received a structured epidemiological assessment of common mental disorders (CMD), ADHD, psychotic symptoms, alcohol and substance abuse, accompanied by additional sections on socio-demographic data, life events, social networks, social supports, disability/activities of daily living, quality of life, use of health services and service use, adapted from the adult psychiatric morbidity schedule (Health and Social Care Information Centre, 2009) used in the UK mental health survey programme.

Demographic information collected included age, sex, ethnicity, marital status and household status (head, spouse or other). Socio-economic factors assessed included employment status, education attainment, economic assets and type of housing.

ADHD was assessed by the WHO Adult ADHD Self-Report Scale (ASRS) Screener (Kessler *et al*. 2007), for each of the six questions, never = 0, rarely = 1, sometimes = 2, often = 3 and very often = 4. This gives a total ASRS score of 0–24. Cases are those with scores of 14 and above. This approach and cut off are recommended by Kessler *et al*. (2007) who examined the validity of the ASRS, tried out three ways of scoring the ASRS and concluded that the most robust approach was to use the full 0–4 response scale for each item to obtain a summary score of 0–24, and that the optimal cut off is a threshold of 13/14, consistently outperforming other approaches including the 0–6 approach, and so recommended it for the future use of the ASRS. Kessler *et al*. (2007) also calculated a range of positive predictive values for each stratum of population prevalence, and found a strong concordance with clinician diagnoses, with an area under the receiving operating characteristic curve of 0.90.

Ramos *et al*. (2009) recommended a cut off of 11/12 which achieved a positive predictive value 91.6% and a negative predictive value 96.5%, in a Spanish outpatient sample. As the Kessler recommendation of a cut off 13/14 was derived from a community sample, we considered that was the most appropriate to use for this study, and note that this approach has also been recently used by Das *et al*. (2014).

CMD were assessed by the Clinical Interview Schedule-Revised (CIS-R) (Lewis *et al*. 1992), a gold standard instrument for use by lay interviewers to assess psychopathology in community settings. It has been widely used in high (Meltzer *et al*. 1995; Jenkins *et al*. 1997*a*, *b*, Singleton *et al*. 2003) and low income countries (Araya *et al*. 2001; Wickramasinghe *et al*. 2002; Patel & Mann, 1997; Patel *et al*. 2006) including Tanzania (Ngoma *et al*. 2003; Jenkins *et al*. 2010*c*) and Kenya (Jenkins *et al*. 2012*a*). The CIS-R measures the presence of 14 symptom-types in the preceding month and the frequency, duration and severity of each symptom in the past week. Scores, taken together with algorithms based on the International Classification of Diseases (ICD)-10 (WHO, 1993), provide diagnoses of depressive episode (mild, moderate or severe), obsessive compulsive disorder, panic disorder, phobic disorder, generalised anxiety disorder and mixed anxiety/depressive disorder. For the purpose of the current paper however, a score of 12 or more across the 14 sections of the survey was considered an indication of ‘any CMD’, as used in other CIS-R studies (Lewis *et al*. 1992; Jenkins *et al*. 1997*a*, *b*).

Respondents were given a list of 11 different stressful life events and asked to say which, if any, they had experienced in the last 6 months. The list included health risks (serious illness, injury or assault to self or close relative), loss of a loved one (death of a relative; death of a close friend), relationship difficulties (separation or divorce; serious problem with a close friend or relative); income instability (being made redundant or sacked; having looked for work for over a month; loss of the equivalent of 3 months income) and legal problems (problems with the police involving a court experience; something of value lost or stolen). The list was developed for the British psychiatric morbidity survey (Meltzer *et al*. 1995; Singleton *et al*. 2003) programme, and slightly modified for the east African context (Jenkins *et al*. 2010*c*). Scores were grouped into ‘none’, ‘one’, ‘two’ and ‘three or more’ life events and were also analysed by category.

Perceived lack of social support was assessed from respondents’ answers to seven questions which were used in the 1992 Health Survey for England (Breeze *et al*. 1994), and the Office of National Statistics (ONS) Surveys of Psychiatric Morbidity (Meltzer *et al*. 1995; Singleton *et al*. 2003). The seven questions take the form of statements that individuals could say were not true, partly true or certainly true for them in response to the question ‘There are people I know who: (i) do things to make me happy; (ii) who make me feel loved; (iii) who can be relied on no matter what happens; (iv) who would see that I am taken care of if I needed to be; (v) who accept me just as I am; (vi) who make me feel an important part of their lives; and (vii) who give me support and encouragement’. Results were categorised into no, moderate or severe lack of perceived social support.

Social network size was assessed by respondents answers to three questions which have also been used in the ONS surveys of Psychiatric morbidity, namely (i) how many adults who live with you do you feel close to, (ii) how many relatives aged 16 or over who do not live with you do you feel close to, (iii) how many friends or acquaintances who do not live with you would you describe as close or good friends. Responses were added into a total social network score.

Specific questions were also asked about caring responsibilities (do you give care due to long term physical or mental disorder or disability? And if yes, time spent giving care in a week); about growing up with one natural parent or two until age 16; and about spending time in an institution before the age of 16.

### Statistical analysis

We examined the prevalence of self-reported ADHD symptoms, CMD, individual diagnostic categories, individual non-psychotic symptoms and of alcohol consumption, hazardous drinking and substance abuse. The estimated prevalence of ADHD self-reported symptoms was examined by calculating the prevalence of ASRS scores of 14 and above (Kessler *et al*. 2007) and reported as proportion of individuals with a 14+ score. Risk factors for ADHD were examined using a logistic regression model with results from the unadjusted analysis reported. Factors considered significant at the bivariate analysis level have been included in the multivariate analysis with odds ratios (ORs) and their 95% confidence interval (CI) reported in the adjusted analysis. We used STATA (Statacorp, 2003) to calculate unadjusted and adjusted ORs. Households have been categorised into different socio-economic levels using an index of household assets, constructed applying the principal component analysis procedure, as a proxy indicator for socio-economic status. In developing the asset quintiles, type of house, roofing and walling material, source of water, toilet facility and land have been used. (Moser, 1998; Morris *et al*. 2000).

### Ethics

Ethical approval was granted by the Kings College London (KCL) and KEMRI boards of research ethics respectively, and by the KEMRI/Walter Reed Project to conduct the study in households within Maseno area, which is part of the Kombewa HDSS.

Informed written and witnessed consent was asked of heads of sampled households, and then of sampled participants to take part in the study.

## Results

A total of 1190 households were selected, and 1158 participants consented to the study while 32 refused to participate in the study interviews, giving a response rate of 97.3%.

Table 1 shows the prevalence of ADHD (using a cut ASRS score of 14+). The prevalence of people with ASRS scores of 14 or more is 13.1%.

**Table 1.** Prevalence of ADHD (ASRS 14+) and its relationship with socio-demographic and psychosocial risk factors (univariate odds ratio)

Factors		*N*	Prevalence: *n* (%)	Unadjusted OR (95% CI)	*p* value
ADHD		1147	150 (13.1)		
Sex	Male	602	73 (12.1)	1	–
	Female	545	77 (14.1)	1.2 (0.85–1.68)	0.316
Age group	<30 years	281	37 (13.2)	1	–
	30–60 years	449	59 (13.1)	1.0 (0.64–1.55)	0.992
	>60 years	171	27 (15.8)	1.2 (0.72–2.12)	0.439
Household size	≤6 people	567	88 (15.5)	1	–
	>6 people	580	62 (10.7)	0.7 (0.46–0.92)	0.016
Marital Status	Married/cohabiting	715	90 (12.6)	1	–
	Single	183	19 (10.4)	0.8 (0.48–1.36)	0.416
	Widowed/divorced	248	40 (16.1)	1.3 (0.89–2.00)	0.161
Education	None	131	19 (14.5)	1	–
	Primary	625	89 (14.2)	1.0 (0.57–1.67)	0.937
	Secondary	320	33 (10.3)	0.7 (0.37–1.24)	0.208
	Post-secondary	71	9 (12.7)	0.9 (0.37–2.00)	0.720
Employment status	Unemployed	563	86 (15.3)	1	–
	Self-employed	485	52 (10.7)	0.7 (0.46–0.96)	0.030
	Employed	99	12 (12.1)	0.8 (0.40–1.46)	0.416
	Positive	269	32 (11.9)	0.8 (0.55–1.30)	0.456
Asset groups	Lowest, Q1	404	44 (10.9)	1	–
	Q2	409	44 (10.8)	1.0 (0.63–1.54)	0.951
	Highest, Q3	334	62 (18.6)	1.9 (1.23 to 2.83)	0.003
Life events	0–1	361	25 (6.9)	1	–
	2–3	477	67 (14.1)	2.2 (1.36–3.55)	0.001
	4 or more	309	58 (18.8)	3.1 (1.89–5.10)	<0.001
Perceived lack of social support	No lack: 0	3	2 (66.7)	1	–
	Moderate lack: 1–7	313	40 (12.8)	0.1 (0.01–0.83)	0.035
	Severe lack: 8+	828	107 (12.9)	0.1 (0.01–0.83)	0.034
Total social group size	3 or less	144	24 (16.7)	1	–
	4–8	519	53 (10.2)	0.6 (0.34–0.96)	0.034
	9 or more	481	72 (15.0)	0.9 (0.53–1.46)	0.620
Presence of CMD	No	1028	122 (11.9)	1	–
	Yes	119	28 (23.5)	2.2 (1.44–3.63)	<0.001
Carer for >4 h	No	26	2 (7.7)	1	–
	Yes	171	20 (11.7)	1.6 (0.35–7.24)	0.549
Spent time at institution <16 years	No	920	124 (13.5)	1	–
	Yes	220	25 (11.4)	1.4 (0.75–2.68)	0.279
Did not live continuously with both natural parents until age 16	No	964	123 (12.8)	1	–
	Yes	176	26 (14.8)	1.2 (0.75–1.87)	0.467
Hazardous drinking	No	1074	140 (13.0)	1	–
	Yes	73	10 (13.7)	1.1 (0.53–2.11)	0.871
Psychotic symptoms	No	963	123 (12.8)	1	–
	Yes	156	25 (16.0)	1.3 (0.82–2.08)	0.267

Table 1 also shows the risk factors associated with ASRS scores of 14 and above, giving ORs, CIs and *p* value.

Odds of ADHD were significantly increased in those with higher assets (OR 1.9, *p* = 0.003), those with more life events (OR 2.2, *p* = 0.001 for those with 2–3 life events and OR 3.1, *p* < 0.009 for 4 or more life events), and were significantly reduced in the self-employed (OR 0.7, *p* = 0.03), those living in larger households (OR 0.7, *p* = 0.016), and those living with a lack of social support (OR 0.1, *p* = 0.035 and OR 0.1, *p* = 0.034 for a moderate lack and severe lack, respectively), and those living with large social group size of 4–8 (OR 0.6, *p* = 0.034) at the bivariate level of analysis.

Table 2 shows the adjusted analysis for risk factors associated with ADHD.

**Table 2.** Factors significantly associated with ADHD, using logistic regression analysis to provide adjusted odds ratio, adjusted for all variables found to be significant at the bivariate level

Factors		Adjusted OR* (95% CI)	*p* value
Household size	>6 people	0.8 (0.56–1.21)	0.323
Employment status	Self-employed	0.7 (0.48–1.05)	0.088
	Employed	0.9 (0.45–1.73)	0.722
Asset Quintiles	Q2 = medium	1.0 (0.64–1.59)	0.968
	Q3 = highest	1.7 (1.07–2.61)	0.023
Life events	2–3	2.4 (1.46–4.00)	0.001
	4 or more	2.6 (1.52–4.39)	<0.001
Perceived lack of social support	Moderate lack: 1–7	0.1 (0.01–1.75)	0.128
	Severe lack: 8+	0.2 (0.01–2.15)	0.173
Total social group size	4–8	0.8 (0.45–1.38)	0.408
	9 or more	1.1 (0.66–1.95)	0.655
Any CMD		2.3 (1.39–3.71)	0.001

* variables identified as univariate predictors of ADHD

The variables for which the outcomes have been adjusted (with *p* value ≤0.05) are household size, employment status, asset quintiles, life events, perceived lack of social support, total social group size and any CMD.

In the adjusted analysis (with factors considered significant in the unadjusted analysis), the self-employed (OR 1.7, *p* = 0.088), those with higher assets (OR 1.7, *p* = 0.023), those with life events (OR 2.4, *p* = 0.001 and OR 2.6, *p* < 0.001 for those with 2–3 life events and 4 or more life events respectively), and those with CMD (OR 2.3, *p* = 0.001) are considered to be predictors/risk factors for ADHD.

The *p* value for the adjusted model, using the Likelihood Ratio test is <0.005.

## Discussion

This is the first household survey of Adult ADHD in Kenya, and the second in sub-Saharan Africa that we have been able to find. This survey of adult ADHD undertaken as part of a mental health epidemiological survey of a household population in Maseno district, Nyanza province in Kenya found that the overall prevalence of ASRS scores of 14 and above was 13.1%.

A range of methods have been used to measure ADHD in the research literature, some using questionnaire screens, some using full clinical diagnostic interview and some using both approaches to provide validation data for the screening instrument. Whenever such validation studies are done, the prevalence rates of ADHD confirmed by diagnostic interview are lower than those identified by the screening instrument. Willcut (2012) in a meta-analytic review of 86 studies of children and adolescents, and 11 studies of adults with ADHD using DSM-IV criteria, found that point prevalence rates of ADHD varied from 4.0 to 13.3 in the meta analysis depending on the specific procedures used to combine information from multiple rates and measure functional impairment. When full clinical diagnostic criteria are used, he found that the overall prevalence of ADHD fell within the range of 5.9–7.1% in children and adolescents and 5% in adults. Faraone & Biederman (2005) administered a semi- structured clinical interview, based on DSM-IV diagnostic criteria for ADHD, to a telephone sample of 966 randomly selected adults in the USA, and estimated prevalence rates of 2.9% for Narrow ADHD and 16.4% for Broad ADHD.

We used the ASRS but we did not validate the ASRS in this population with a gold standard clinical interview to confirm cases relative to full diagnostic ICD or DSM criteria, but rather used the cut off recommended by Kessler *et al*. (2007). Direct comparisons of our results can therefore best be made with other studies which used the same instrument and the same scoring method. Two studies meet this criterion. Firstly, Kessler *et al*. (2007) used a personal ASRS screen, with a cut off 13/14, followed by a telephone clinical reappraisal interview to a national sample in the USA, and found a weighted prevalence of 8.4% of clinical adult ADHD. Secondly, Das *et al*. (2014) also used the ASRS and found 2.2% of the older age cohort scored equal or above the ASRS cut-off score of 14 compared with 6.2% of middle aged adults. Kessler *et al*. (2007) classified cases in partial remission as not cases which is a conservative approach. Cases in partial remission are quite common (Faraone & Biederman, 2005), and so if they had been included, their prevalence rate would have been higher.

Prevalence rates found in other household studies of adult populations range from 12.2% for Nigeria using the Barkley Adult ADHD Rating Scale (Okhakhume & Oluwefaemi, 2014) to a pooled prevalence of 2.5% (CI 2.1–3.1) in a meta-analysis of western studies (Simon *et al*. 2009), which had used a variety of sample designs and instruments. Simon *et al*. (2009) concluded that this pooled prevalence may be too restrictive because the DSM-IV criteria underestimate ADHD in adults.

The World Mental Health Survey in 10 countries (Belgium, France, Germany, Italy, Netherlands and Spain in Europe, the USA, Colombia, Mexico and the Lebanon.) used the ASRS with O-6 scoring (Fayyad *et al*. 2007). The estimated prevalence was based on multiple imputations, and the imputation model was based on a clinical calibration conducted only in the USA and not in the other countries studies. It found an average prevalence of 3.4% (range 1.2–7.3%) across the 10 countries in the World Mental Health Survey which did not include any from Africa.

The British national household survey (Health and Social Care Information Centre 2007) found that 8.3% screened positive for ADHD, using the ASRS screener and the O-6 scoring method, with a cut off of 3/4.

There have also been a few epidemiological studies of ADHD in college students. One study of Kenyan medical students (Atwoli *et al*. 2011) used the 0–6 scoring method for the ASRS screener and found a prevalence of 23.7% of ADHD symptoms in Kenyan medical students, with 8.7% satisfying DSM-IV criteria for an ADHD diagnosis, while De Paul *et al*. (2001) found rates of ADHD up to 8.1% in Italy, New Zealand and the USA (De Paul *et al*. 2001).

Atwoli *et al*. (2011) argue that, as well as methodological issues posed by screening instruments being a relevant issue in comparing survey results, it is possible that rates of ADHD are actually much higher in low income countries than in the west. Indeed it has also been argued that ADHD may carry some evolutionary advantage in nomadic tribes, (Eisenberg *et al*. 2008; Eisenberg & Campbell, 2011) and so previous natural selection of genes linked to ADHD symptoms may go some way towards explaining higher rates found so far in low income countries in Africa.

We did not find a gender difference in ADHD prevalence, similar to the lack of gender difference in other adult studies (Fayyad *et al*. 2007; Health and Social Care Information Centre, 2009; Simon *et al*. 2009; Okhakhume & Oluwefaemi, 2014) of ADHD. However we did find significantly increased odds in the adjusted analysis in the self-employed (OR 1.7, *p* = 0.088), those with higher assets (OR 1.7, *p* = 0.023), those with life events (OR 2.4, *p* = 0.001 for those with 2–3 life events and OR 2.6, *p* < 0.001 for those with 4 or more life events), and those with CMD (OR 2.3, *p* = 0.001).

The relationship between ADHD and self-employment is puzzling. It would be expected that people with ADHD might be more likely to choose self-employment, where it may be more possible to choose niches that fit their strengths and energies, and where ADHD is a clear benefit rather than a disability (Eisenberg & Campbell, 2011). On the other hand it may be that being in self-employment enables ADHD symptoms to be less prominent than they would be in the confines of more structured employment.

We have not found comparable studies of adult ADHD and assets, but our finding of an association of ADHD symptoms with high assets stands in contrast to the British National Survey (Health and Social Care Information Centre, 2009) which found odds were higher in those with low household income, and also in contrast to Faraone & Biederman (2005) which found associations between ADHD and lower levels of employment status, while Willcut (2012) found an association between ADHD and low socio-economic status in some but not all studies. Das *et al*. (2012) found significant negative associations (*p* < 0.01) with employment and financial stress, and the British mental health survey found an association of ADHD with unemployment and economic inactivity (Health and Social Care Information Centre, 2009).

We did not find an association with educational level, unlike Faraone & Biederman (2005) who found an association with lower levels of education, and the British National Survey which also found an association with those who had no educational qualifications (Health and Social Care Information Centre, 2009). This is a complex question to examine as it is possible that the screener works better in higher educated groups and not so well in those with less sophisticated use of language, and so the relationship of ADHD with educational level needs further exploration.

We did not find significant comorbidity with psychotic symptoms or with hazardous drinking, and indeed a diagnosis of ADHD is precluded in the presence of psychosis in DSM-IV. (Willcut, 2012) The comorbidity we found with CMD was also found in the World Mental Health Survey (Fayyad *et al*. 2007); in the British survey (Health and Social Care Information Centre, 2009) which found comorbidity with attempted suicide, panic disorder, phobia, post traumatic stress disorder (PTSD), Psychosis, depressive episode and antisocial personality disorder but not with alcohol or drug dependency; and in the study of Das *et al*. (2012) who found strong positive correlations between symptoms of ADHD and depression/anxiety. The associations we found with increased unpleasant life events and with CMD indicate the vulnerability and disadvantage still experienced by people with ADHD in Africa as well as in western countries (Ginsberg *et al*. 2014). Understanding comorbidity is important because these comorbid conditions often clinically overshadow the underlying ADHD syndrome (Cumyn *et al*. 2007).

### Strengths of study

The strengths of the study are the use of a health and demographic surveillance site for the random sample of households, the high response rate and the systematic approach to the clinical and socio-demographic assessments. The population in the surveillance site is regularly monitored by field staff who visit each household bi-annually to capture health and demographic information (birth rates, death rates, causes of death, pregnancies, immunization status, in-and out-migrations, etc.). Various studies nested on the DSS platform take advantage of the sampling frame inherent in the HDSS, whether at individual, household/compound or regional levels. This familiarity with survey procedures is likely to have been influential in the achievement of a high response rate. The ASRS proved reasonably straightforward to include in the overall research interview with questions specified on the PDA, and responses directly recorded to the PDA. The questions were available on the PDA in English, Kiswahili and Luo (the main local language). Our research interviewers were trained health workers who lived locally and who were therefore familiar with the local languages. It was very important to ensure that the sample was properly random, and therefore we drew on the supervision of the demographic surveillance site staff as well as the project manager to ensure that the randomisation procedures were followed in the identification of households and the identification of one adult per household

### Limitations of study

Our study findings were limited by the fact that we did not validate the ASRS with a clinical diagnostic interview in this population. This is important to do as the wording of the ASRS may not always be understood in all populations and therefore accuracy of the screener is not clear until validated against diagnostic interview. If accurate estimates of ADHD are to be generated, it is essential to do a study with validation against the gold standard of diagnostic interview in a random subsample.

The validity of the ASRS screener has been examined in the USA (Kessler *et al*. 2006, 2007) but not in Kenya, and so future studies in Kenya and indeed elsewhere need to compare the results of the ASRS screener with semi-structured standardised research clinical assessments.

We used the cut off of 13/14 as recommended by Kessler *et al*. (2007) from a USA sample. Although Ramos-Quiroga (2009) as subsequently recommended a lower cut off 11/12, this was based on a Spanish outpatient sample, so we still consider use of the cut off 13/14 as recommended by Kessler *et al*. (2007) remains the most appropriate and reasonably conservative approach for community samples in Africa pending local validity studies.

We consider that our findings of the relatively high prevalence of ASRS (14+) scores in Kenya indicate the importance of further research on this important condition both in Kenya and in other low and high-income countries, in order to have a better understanding of overall global mental health and illness. Since ADHD is essentially a clinical diagnosis, it is important in future studies in both high and low income countries to combine the ASRS screen with a clinical interview to obtain more information on the optimum cut off screening score in each population in which the screen is used, and the methodological challenges might inform the population measuring and surveillance of this condition in other settings, including high-income ones. The use of a demographic surveillance site as a sample frame where possible confers a number of advantages to the implementation of epidemiological surveys, particularly a relatively high response rate, which reduces the effects of non-response bias in the findings.

### Conclusions

The study demonstrates the magnitude of ADHD symptoms, as measured by ASRS screener scores of 14 and over, as a public health issue in Kenya, which is relevant for health worker training about detection and management. The study also indicates the importance of further research into measurement and surveillance of adult prevalence rates in both high and low income countries using a screening instrument to enable the coverage of large sample sizes together with a gold standard clinical assessment of a random subsample to assess validity of the screener in that population. It is also crucial to conduct further exploration of the complexity of associated risk factors, which may vary in different contexts, and between high and low income countries.

## References

[ref1] Araya R, Rojas G, Aritsch R, Acuna J, Lewis G (2001). Common mental disorders in Santiago, Chile: prevalence and socio-demographic correlates. British Journal of Psychiatry 178, 228–233. 11230033 10.1192/bjp.178.3.228

[ref2] Atwoli L, Owiti P, Manguro G, Ndambuki D (2011). Attention deficit hyperactivity disorder symptom self-report among medical students in Eldoret, Kenya. African Journal of Psychiatry 2011, 286–289. 22038426 10.4314/ajpsy.v14i4.5

[ref3] Biederman J (1998). Attention deficit-hyperactivity disorder-a life span perspective. Journal of Clinical Psychiatry 59(Suppl. 7), 4–16. 9680048

[ref4] Breeze E, Maidment A, Bennett N, Flatley J, Carey S (1994). Health Survey for England, 1992. HMSO: London, UK.

[ref5] Cumyn L, Kolar D, Keller A, Hechtman L (2007). Current issues and trends in the diagnosis and treatment of adults with ADHD. Expert Review of Neurotherapeutics 7, 1375–1390. doi: 10.1586/14737175.7.10.1375 17939773

[ref6] Das D, Cherbuin N, Butterworth P, Anstey KJ, Easteal S (2012). A population-based study of attention deficit/hyperactivity disorder symptoms and associated impairment in middle-aged adults. PLoS ONE 7, e14. doi: 10.1371/journal.pone.0031500. Epub 2012 Feb 8. PMC3275565 22347487

[ref7] Das D, Cherbuin N, Easteal S, Anstey KJ (2014). Attention deficit/hyperactivity disorder symptoms and cognitive abilities in the late-life cohort of the PATH through life study. PLoS ONE 9, e14. doi: 10.1371/journal.pone.0086552. eCollection 2014. PMC3904910 24489743

[ref8] De Paul GJ, Schaughenency EA, Weyandt LL, Tripp G, Kisener J, Ota K (2001). Self report of ADHD symptoms in university students: cross gender and cross national prevalence. Journal of Learning Disabilities 34, 370–379. 15503581 10.1177/002221940103400412

[ref9] Eisenberg D, Campbell B (2011). The evolution of ADHD. *San Francisco Medicine* October, pp. 21–22.

[ref10] Eisenberg DTA, Campbell B, Gray PB, Sorenson MD (2008). Dopamine receptor genetic polymorphisms and body composition in undernourished pastoralists: an exploration of nutrition indices among nomadic and recently settled Ariaal men of northern Kenya. BMC Evolutionary Biology 8, 173, 1471–2148. 10.1186/1471-2148-8-173 PMC2440754 18544160

[ref11] Faraone SV, Biederman J (2005). What is the prevalence of adult ADHD? Results of a population screen of 966 adults. Journal of Attention Disorders 9, 384–391. 16371661 10.1177/1087054705281478

[ref12] Fayyad J, De Graaf R, Kessler R, Alonso J, Angermeyer M, Demyttenaere K, De Girolamo G, Haro JM, Karam EG, Lara C, Demyttenaere K, Lepine JP, Ormel J, Posada-Villa J, Zaslavsky AM (2007). Cross-national prevalence and correlates of adult attention-deficit hyperactivity disorder in. British Journal of Psychiatry 190, 402–409. Doi: 10.1192. 17470954 10.1192/bjp.bp.106.034389

[ref13] Ginsberg Y, Beusterien KM, Amos K, Jouiseelin C, Asherson P (2014). The unmet needs of all adults with ADHD are not the same: a focus on Europe. Expert Review of Neurotherapeutics 14, 799–812. 24894408 10.1586/14737175.2014.926220

[ref14] Health and Social Care Information Centre (2009). Adult Psychiatric Morbidity Survey 2007 Social care Statistics. http://www.hscic.gov.uk/pubs/psychiatricmorbidity07

[ref15] Jenkins R, Bebbington P, Brugha T, Farrell M, Gill B, Lewis G, Meltzer H, Petticrew M (1997 *a*). The national psychiatric morbidity surveys of Great Britain—strategy and methods. Psychological Medicine 27, 765–774. 9234455 10.1017/s003329179700531x

[ref16] Jenkins R, Kiima D, Njenga F, Okonji M, Kingora J (2010 *a*). Integration of mental health into primary care in Kenya. World Psychiatry 9, 118–120. 20671901 10.1002/j.2051-5545.2010.tb00289.x PMC2911092

[ref17] Jenkins R, Kiima D, Okonji M, Njenga F, Kingora J (2010 *b*). Integration of mental health in primary care and community health working in Kenya: context, rationale, coverage and sustainability. Mental Health in Family Medicine 7, 37–47. 22477921 PMC2925163

[ref18] Jenkins R, Lewis G, Bebbington P, Brugha T, Farrell M, Gill B, Meltzer H (1997 *b*). The national psychiatric morbidity surveys of Great Britain—initial findings from the household survey. Psychological Medicine 27, 775–790. 9234456 10.1017/s0033291797005308

[ref19] Jenkins R, Mbatia J, Singleton N, White B (2010 *c*). Common mental disorders and risk factors in urban Tanzania. International Journal of Environmental Research and Public Health 7, 2543–2558. 20644689 10.3390/ijerph7062543 PMC2905566

[ref20] Jenkins R, Njenga F, Okonji M, Kigamwa P, Baraza M, Ayuyo J, Singleton N, McManus S, Kiima D (2012 *a*). Prevalence of common mental disorders in a rural district of Kenya, and socio-demographic risk factors. International Journal of Environmental Research and Public Health 9, 1810–1819; doi: 10.3390/ijerph9051810 22754474 PMC3386589

[ref21] Jenkins R, Njenga F, Okonji M, Kigamwa P, Baraza M, Ayuyo J, Singleton N, McManus S, Kiima D (2012 *b*). Psychotic symptoms in Kenya – prevalence and risk factors, including their relationship with common mental disorders. International Journal of Environmental Research and Public Health 9, 1748–1756. 22754470 10.3390/ijerph9051748 PMC3386585

[ref22] Jenkins R, Othieno C, Okeyo S, Aruwa J, Kingora J, Jenkins B (2013 *b*). Health system challenges to integration of mental health delivery in primary care in Kenya-perspectives of primary care health workers. BMC Health Services Research 13, 368. 24079756 10.1186/1472-6963-13-368 PMC3852631

[ref23] Jenkins R, Othieno C, Okeyo S, Aruwa J, Wallcraft J, Jenkins B (2013 *c*) Exploring the perspectives and experiences of health workers at primary health facilities in Kenya following training. International Journal of Mental Health Systems 7, 6. 23379737 10.1186/1752-4458-7-6 PMC3599922

[ref24] Jenkins R, Othieno C, Okeyo S, Kaseje D, Aruwa J, Oyugi H, Bassett P, Kauye F (2013 *a*). Short structured general mental health in service training programme in Kenya improves patient health and social outcomes but not detection of mental health problems – a pragmatic cluster randomised controlled trial. International Journal of Mental Health Systems 7, 25. 24188964 10.1186/1752-4458-7-25 PMC4174904

[ref25] Kenya Bureau of Statistics (2011). http://www.prb.org/pdf11/kenya-population-data-sheet-2011.pdf

[ref26] Kessler R, Adler L, Barkely R, Biederman J, Conners CK, Demler O, Faraone SV, Greenhill LL, Howes MJ (2006). The prevalence and correlates of adult ADHD in the Unites States: results from the national comorbidity survey replication. American Journal of Psychiatry 163, 716–723. 16585449 10.1176/appi.ajp.163.4.716 PMC2859678

[ref27] Kessler RC, Adler L, Gruber MJ, Sarawate CA, Spencer T, Van Brunt DL (2007). Validity of the World Health organisation Adult ADHD Self Report Scale (ASRS) Screener in a representative sample of health plan members. International Journal of Methods in Psychiatric Research 16, 52–65. 17623385 10.1002/mpr.208 PMC2044504

[ref28] Kiima D, Jenkins R (2010). Mental health policy in Kenya – an integrated approach to scaling up equitable care for poor populations. International Journal of Mental Health Systems 4, 19. 20584266 10.1186/1752-4458-4-19 PMC2907308

[ref29] Kiima D, Njenga F, Shah A, Okonji M, Ayuyo J, Baraza M, Parker E, Jenkins R (2009). Attitudes to depression among community health workers in Kenya. Epidemiologica e Psychiatrica 18, 352–356. 20170051

[ref30] Kiima DM, Njenga FG, Okonji MM, Kigama PA (2004). Kenya mental health country profile. International Review of Psychiatry 16, 48–53. 15276937 10.1080/09540260310001635096

[ref31] Kimanga DO, Ogola S, Umuro M, Nganga A, Kimondo L, Mureithi P, Muttunga J, Waruiru W, Mohammed I, Sharrif S, De Cock KM, Kim AA (2014). Prevalence and incidence of HIV infection, trends, and risk factors among persons aged 15–64 years in Kenya: results from a nationally representative study. Journal of Acquired Immune Deficiency Syndrome Jan 17 66 (Suppl 1), S13–26. Abstract available at http://www.ncbi.nlm.nih.gov/pubmed/24445338 10.1097/QAI.0000000000000124 PMC4794992 24445338

[ref32] Kooij SJJ, Bejerot S, Blackwell A, Caci H, Casas-Brugué M, Carpentier PJ, Asherson P (2010). European consensus statement on diagnosis and treatment of adult ADHD: the European Network Adult ADHD. BMC Psychiatry 10, 67. doi: 10.1186/1471-244X-10-67. 20815868 PMC2942810

[ref33] Lewis G, Pelosi A, Araya RC, Dunn G (1992). Measuring psychiatric disorder in the community: a standardised assessment for use by lay interviewers. Psychological Medicine 22, 465–489. 1615114 10.1017/s0033291700030415

[ref34] Mannuzza S, Klein RG, Bessler A, Mallov P, LaPadula M (1993). Adult outcome of hyperactive boys: educational achievement , occupational rank, and psychiatric status. Archives of General Psychiatry 50, 565–576. 8317950 10.1001/archpsyc.1993.01820190067007

[ref35] Meltzer H, Gill B, Petticrew M, Hinds K (1995). *OPCS Survey of Psychiatric Morbidity: Report 1. The Prevalence of Psychiatric Morbidity among Adults Ages 16–64 Living in Private Households in Great Britain*. HMSO: London, UK.

[ref36] Morris SS, Carletto C, Hoddinott J, Christiaensen LJM (2000). Validity of rapid estimates of household wealth and income for health surveys in rural Africa. Journal of Epidemiology and Community Health 54, 381–387. 10814660 10.1136/jech.54.5.381 PMC1731675

[ref37] Moser C (1998). The asset vulnerability framework: reassessing urban poverty reduction strategies. World Development 26, 1–19.

[ref38] Muga F, Jenkins R (2008 *a*). Public perceptions, explanatory models and service utilisation regarding mental illness and mental health care in Kenya. Social Psychiatry and Psychiatric Epidemiology 43, 469–476. 18427705 10.1007/s00127-008-0334-0

[ref39] Muga F, Jenkins R (2008 *b*). Training, attitudes and practice of district health workers in Kenya. Social Psychiatry and Psychiatric Epidemiology 43, 477–482. 18327522 10.1007/s00127-008-0327-z

[ref40] Mugawebster F, Jenkins R (2010). Health care models guiding mental health policy in Kenya 1965–1997. International Journal of Mental Health Systems 4, 9. 20426855 10.1186/1752-4458-4-9 PMC2872652

[ref41] Ngoma MC, Prince M, Mann A (2003). Common mental disorders among those attending primary health clinics and traditional healers in urban Tanzania. British Journal of Psychiatry 183, 349–355. 14519614 10.1192/bjp.183.4.349

[ref42] Okonji M, Njenga F, Kiima D, Ayuyo J, Kigamwa P, Shah A, Jenkins R (2008). Traditional health practitioners and mental health in Kenya. International Psychiatry 5, 46–48. PMC6734814 31507940

[ref43] Okhakhume AS, Oluwefaemi AA (2014). Self reported symptoms of adult ADHD among the general population in Nigeria. Kenya Journal of Educational Planning, Economics and Management 7, 1–23.

[ref44] Oluoch T, Mohammed I, Bunnell R (2011). Correlates of HIV Infection Among Sexually Active Adults in Kenya: ANational Population-Based Survey. *The Open AIDS Journal,* 2011, *5,* 125–134. *The Millennium Development Goals Repor*t; United Nations: New York, NY, USA, 2007. 10.2174/1874613601105010125 PMC3257551 22253668

[ref45] Othieno C, Jenkins R, Okeyo S, Aruwa J, Wallcraft J, Jenkins B (2013). Perspectives and concerns of clients at primary health care facilities involved in evaluation of a national mental health training programme for primary care in Kenya. International Journal of Mental Health Systems 7, 5. 23343127 10.1186/1752-4458-7-5 PMC3576266

[ref46] Patel V, Kirkwood BR, Pednekar S, Weiss H, Mabey D (2006). Risk factors for common mental disorders in women: population-based longitudinal study. British Journal of Psychiatry 189, 547–555. 17139040 10.1192/bjp.bp.106.022558

[ref59] Patel V, Mann A (1997). Etic and emic criteria for non-psychotic mental disorder: a study of the CISR and care provider assessment in Harare. Social Psychiatry and Psychiatric Epidemiology 32, 84–89. Int. J. Environ. Res. Public Health 2010, 7 2556. 9050349 10.1007/BF00788925

[ref47] Polanczyk G, delima MS, Horta BL, Biederman J, Rohdeee LA (2007). The worldwide prevalence of ADHD: a systematic review and meta-regression analysis. American Journal of Psychiatry 164, 2581–90. 10.1176/ajp.2007.164.6.942 17541055

[ref48] Ramos-Quiroga JA, Daigre C, Valero S, Bosch R, Gómez-Barros N, Nogueira M, Palomar G, Roncero C, Casas M (2009). Validation of the Spanish version of the attention deficit hyperactivity disorder adult screening scale (ASRS v. 1.1): a novel scoring strategy. Revue Neurologique 48, 449–452. 19396760

[ref49] Ramos-Quiroga JA, Nasillo V, Fernández-Aranda F, Fernández-Arana F, Casas M (2014). Addressing the lack of studies in attention-deficit/hyperactivity disorder in adults. Expert Review of Neurotherapeutics 14, 553–567. doi: 10.1586/14737175.2014.908708 24738746

[ref50] Sifuna P, Oyugi M, Ogutu B, Andagalu B, Otieno A, Owira V, Otsyula N, Oyieko J, Cowden J, Otieno L, Otieno W (2014). Health & Demographic Surveillance System Profile: the Kombewa Health and Demographic Surveillance System (Kombewa HDSS). International Journal of Epidemiology 43, 1097–1104. doi: 10.1093/ije/dyu139 PMC4258789 25009309

[ref51] Simon V, Czobor P, Balint S, Meszaros A and Bitter I (2009). Prevalence and correlates of adult attention deficit hyperactivity disorder-meta analysis. British Journal of Psychiatry 194, 204–211. 19252145 10.1192/bjp.bp.107.048827

[ref52] Singleton N, Bumpstead R, O'Brien M, Lee A, Meltzer H (2003). Psychiatric morbidity among adults living in private households, 2000. International Review of Psychiatry 15, 65–73. 12745312 10.1080/0954026021000045967

[ref53] Statacorp (2003). Stata Statistical Software: Release 11.2. In: College Station TX: StataCorp LP, editor.

[ref54] UNDP Human Development report (2013). http://hdr.undp.org/sites/default/files/Country-Profiles/KEN.pdf

[ref55] United Nations (2007). *The Millennium Development Goals Report*. United Nations: New York, NY, USA.

[ref56] Wickramasinghe SC, Rajapakse L, Abeysinghe R, Prince M (2002). The clinical interview schedule-sinhala version: validation in a community setting in Sri Lanka. International Journal of Methods in Psychiatric Research 11, 169–177. 12459820 10.1002/mpr.134 PMC6878291

[ref57] Willcut EG (2012). The prevalence of DSM IV attention deficit/hyperactivity disorder: a meta analytic review. Neurotherapeutics 9, 490–499. 22976615 10.1007/s13311-012-0135-8 PMC3441936

[ref58] World Health Organization (1993). International Statistical Classification of Diseases and Related Health Problems. Tenth Revision. World Health Organization: Geneva, Switzerland. 3376487

